# Endosymbiotic Bacteria Associated with the Mealy Bug, *Rhizoecus amorphophalli* (Hemiptera: Pseudococcidae)

**DOI:** 10.1155/2014/268491

**Published:** 2014-11-05

**Authors:** Ravikumar Sreekala Sreerag, C. A. Jayaprakas, L. Ragesh, Sasidharan Nishanth Kumar

**Affiliations:** Division of Crop Protection, Central Tuber Crops Research Institute, Sreekariyam, Thiruvananthapuram, Kerala 695 017, India

## Abstract

The mealy bug, *Rhizoecus amorphophalli*, is a menace to the aroid farmers due to the intensive infestation on stored tubers. Spraying of pesticides was able to control this pest but it always left a chance for fungal growth. Bacterial endosymbionts associated with the insects provide several benefits to their host. Since such endosymbionts play a vital role even in the physiology of their host, revealing the types of bacteria associated with mealy bug will give basic information, which may throw light on the management of this noxious pest. The present study is the first to identify bacterial endosymbionts associated with *R. amorphophalli* employing phenotypic characterization and 16S rDNA sequencing. Three culturable bacteria, namely, *Bacillus subtilis*, *Staphylococcus gallinarum*, and *Staphylococcus saprophyticus*, were isolated from *R. amorphophalli*. Moreover, the antibiotic susceptibility tests against the isolated bacteria showed that all the isolates were susceptible to the three antibiotics tested, except cephalexin. Recently, endosymbionts are used as effective biocontrol agents (BCAs) and the present study will stand as a connecting link in identification and effective utilization of these endosymbionts as BCAs for management of *R. amorphophalli*.

## 1. Introduction

Insects exhibit symbiotic relationships with bacteria [1] and they provide several benefits to their host [2]. Essential insect symbionts offer a desirable target for the control of insect pests that rely upon them. The complete elimination of endosymbionts using antibiotics reduces the lifespan of the insect and suppresses the population within a few days or weeks [3].

In the insect pest complex in agricultural ecosystem, mealy bugs (Homoptera: Pseudococcidae) have been rated as a major pest, since they play a dual role as pest and vector in field crops [4, 5]. Mealy bugs suck sap from their host and the nutrient deficient plant becomes stunted and distorted and shows reduced vigor [6, 7]. The infestation of the mealy bug,* Rhizoecus amorphophalli* Betrem, on stored tubers of aroids and yams is a major concern among the tuber crop farmers. Tuber crops including aroids and yams play a vital role in food security [8, 9] and are the important staple or subsidiary food for one-fifth of the world population. Mealy bugs suck cell sap from the tubers and the severely infested deformed tubers find no place in market, nor are they accepted for cooking [10]. Mealy bugs multiplication is rapid during the season with high temperature and less humidity and they spread all over the tubers with white powdery mealy substance and disfigure the tubers [11].

Since rapid and effective methods to control insect pests represent one of the many scientific achievements of the twentieth century, use of antibiotics for pest management is a viable option [12]. Elimination of gut bacteria of southern green stink bug,* Nezara viridula* L., using antibiotics reduced the weight of newly emerging adults [13].

Since endosymbionts play a vital role in the physiology of their host, revealing the types of bacteria associated with mealy bug will give basic information, which may throw light on the management of this pest. The present study deals with the isolation and identification of endosymbionts by classical and molecular approach and their antimicrobial activity.

## 2. Materials and Methods

### 2.1. Isolation of Bacteria

Adult females of* R. amorphophalli* collected from the stock culturewere surface sterilized with absolute ethanol and chlorine bleach, were homogenized in sterile 0.9% saline, and were plated directly on nutrient agar media and kept for aerobic overnight incubation at 30°C.

### 2.2. Biochemical Identification of Bacteria

Pure culture of each bacterium for biochemical identification was obtained by streaking the individual colony on a fresh nutrient agar plate and incubated for 24 h at 35°C. Each well separated colony was subjected to the following biochemical tests.

#### 2.2.1. Gram Staining

The gram staining was performed using Hi-Media kit (Hi-Media Laboratories Pvt. Ltd., India) according to the manufacture's protocol. The results were observed using Leica DMLB compound microscope (100x).

#### 2.2.2. Motility

Motility of the isolates was tested using hanging drop method (Hi-Media). Take a clean, scratch-free glass slide. Place 2 drops of culture in the middle of the slide and place a clean cover slip over the drop and examined under microscope.

#### 2.2.3. Carbohydrate Fermentation

Monosaccharide (fructose), disaccharide (sucrose, lactose, and maltose), polyhydric alcohol (mannitol), and hexose (dextrose) were tested. Bromothymol was used as the indicator. Triple sugar-iron agar test (TSI) was also done according to the manufacture's protocol (Hi-Media, Mumbai, India).

#### 2.2.4. IMViC Test

The production of tryptophanase, sufficient amount of alcohol, acetyl methyl carbinol, and citrate utilisation were noted using the indole test, methyl red (MR) test, Voges-Proskauer (VP) test, and citrate utilization test, respectively, according to the protocol of Mackie McCartney [14].

#### 2.2.5. Biochemical Tests for Enzyme Production

Exoenzymes produced were noted using urease test, O-nitrophenyl-beta-D-galactoside (ONPG) (*β*-galactosidase) test, and nitrate reduction test according to the protocol of Mackie McCartney [14].

### 2.3. Pure Culture Maintenance

Pure cultures were maintained by subsequent subculture/transfer in nutrient agar medium every 15 days. Cultures were preserved by freezing at −70°C in 20% glycerol.

### 2.4. Molecular Identification

#### 2.4.1. Genomic DNA Isolation and 16S rDNA Amplification

Total genomic DNA was extracted from bacterial cultures and treated with 100 *μ*L of RNase (Banglore Genei, India) for eliminating the RNA contamination. The bacterial isolates were identified using 16S rDNA gene specific primers (16SP0, 5′-GAAGAGTTTGATCCTGGCTCAG-3′, and 16SP6, 5′-CTACGGCTACCTTGTTACGA-3′) [15]. PCR amplification was carried out in 25 *μ*L reaction volume which included the following components: 5–10 *μ*L of genomic DNA (500 ng), 20 picomoles of each primer, 10 mM Tris HCl (pH-8.3), 50 mM KCl, 2.5 mM MgCl_2_, 0.25 mM of each dNTP, and 0.5 U of Taq DNA polymerase (Fermentas Life Sciences, EU). Amplification was carried out in a thermal cycler (Applied Biosystems, Veriti, USA) with cycling regime—94°C for 4 minutes as initial denaturation followed by 35 cycles of 94°C for 30 seconds, 55°C for 45 seconds, 72°C for 45 seconds, and 72°C for 10 minutes as final extension. The amplified products resolved on 1.2% agarose gel were stained with ethidium bromide (10 *μ*g/mL) and visualized in a gel documentation system (UVP, Analytika Jena, Germany).

#### 2.4.2. Molecular Cloning and Sequencing

PCR products were gel-purified and ligated into T/A cloning vector PTZ57R/T (InsTAclone PCR cloning kit, Fermentas, EU) and transformed into competent DH5*α* cells according to the manufacturer's protocol. The transformed cells were spread on LB agar plates supplemented with X-gal (300 *μ*g/mL), IPTG (120 *μ*g/mL), and ampicillin (100 *μ*g/mL) and incubated at 37°C overnight. Blue/white selection was carried out and the plasmids were isolated from the positive clones using Gene JET Plasmid Miniprep Kit (Fermentas, EU) according to the manufacturer's protocol. Sequencing was carried out in an automated sequencer (ABI Prism 310; Applied Biosystems, USA) using M13 Universal primers in both directions.

#### 2.4.3. Sequence Analysis

The nucleotide sequence obtained was processed to remove low quality reads and transformed into consensus sequences with Geneious Pro software version 5.6. The resulted high quality sequences were analyzed with BLASTn (NCBI; http://www.ncbi.nlm.nih.gov) to confirm the authenticity of the isolate. The sequences of related species and genus were downloaded from the Genbank database and a phylogenetic study was carried out with the program MEGA version 5 [16]. The sequences were aligned using the computer package ClustalW [17] and were analyzed to determine the relationships between isolates by the neighbor-joining method [18] using the Maximum Composite Likelihood model. Bootstrap values were generated using 2000 replicates to infer the robustness of the tree topology.

#### 2.4.4. Antibiotic Susceptibility of Bacteria Isolated from* Rhizoecus amorphophalli*

Antibiotic susceptibility of each isolate was determined by the disk diffusion method [19]. The bacterial cultures maintained in nutrient agar slant at 4°C were subcultured in nutrient broth to obtain the working cultures approximately containing 1 × 10^6^ CFU/mL. Mueller Hinton agar plates (Hi-Media, Mumbai, India) were swabbed with each bacterial strain and the antibiotic disks were placed on the plates. Disks of cephalexin (30 *μ*g/disc) (CP), ciproflax (5 *μ*g/disc) (CFx), endrofloxaxin (10 *μ*g/disc) (Ex), and cefixime (5 *μ*g/disc) (CFIx) were used. Plates were incubated overnight at 37°C. Clear, distinct zone of inhibition was visualized surrounding the disks. The antimicrobial activity was determined by measuring the zone of inhibition expressed in mm. The sensitivity and resistance of each isolate were determined by the criteria of the National Committee for Clinical Laboratory Standards (1997).

## 3. Results

A total of five bacterial strains were successfully isolated from the* R. amorphophalli *and were assigned code numbers as isolates Rhizo 1, Rhizo 2, Rhizo 3, Rhizo 4, and Rhizo 5 for laboratory purposes.

### 3.1. Phenotypic Characters of Isolates

Isolates Rhizo 1, Rhizo 2, and Rhizo 3 showed similar biochemical characters (Table 1). They were gram positive, motile, rod-shaped bacteria. Nutrient agar colonies were circular, entire, and flat. Acid was produced from dextrose and lactose and no acid was produced from sucrose, mannitol, maltose, fructose, and starch. In IMViC test, citrate was positive and indole, methyl red and VP was negative. Being slightly positive to urease and TSI gave acidic slant and acidic butt. Nitrate converted to nitrite.

The isolate Rhizo 4 was motile, gram positive, and cocci shaped. Nutrient agar colonies were irregular, lobate, and creamy yellow in colour (Table 1). Acid was produced from all sugars tested. In IMViC test citrate and methyl red positive, negative to indole and VP. Positive to urease and with TSI gave acidic slant with acidic butt. Nitrate converted to nitrite.

The isolate Rhizo 5 differed from other isolates with small, irregular, and white colonies. They were gram positive and cocci shaped and even though all other isolates were motile, this bacterium was nonmotile. Acid was produced from all sugars tested. IMViC test showed positive to citrate only (Table 1). Nitrate converted to nitrite.

The biochemical characters of all isolates were verified using Bergey's Manual of Systematic Bacteriology Volume 2 (2005) and Bergey's Manual of Systematic Bacteriology Volume 3 (2009). Based on morphological and biochemical characteristics (Table 1), isolates 1, 2, and 3 strains were similar to* Bacillus* sp., isolates 4 and 5 were similar to* Staphylococcus* sp.

Although biochemical tests revealed the preliminary identity of the isolates, for definitive identification 16S rDNA sequencing was performed.

### 3.2. 16S rDNA Analysis

**Figure 1:**
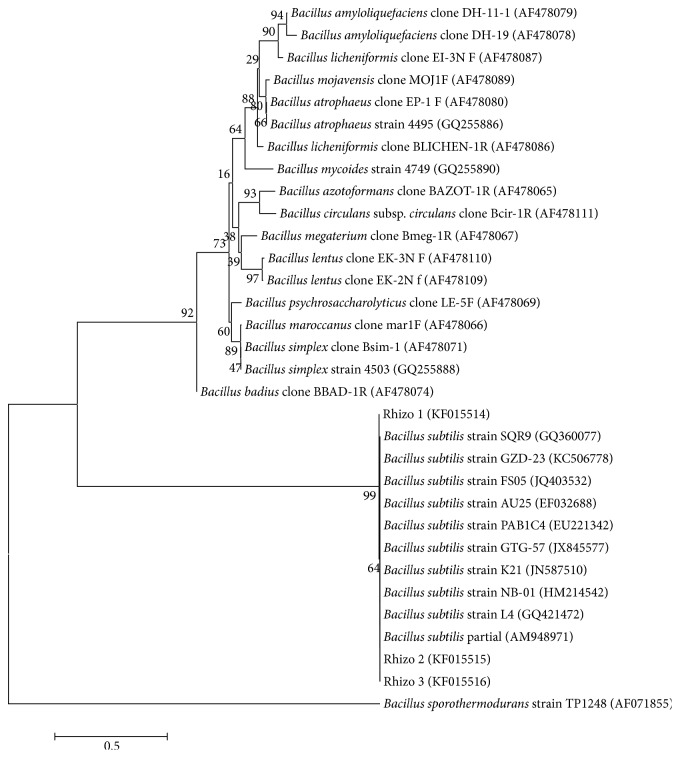
Neighbor-joining (NJ) tree showing the evolutionary relationship of* Bacillus subtilis* (Rhizo 1, Rhizo 2, and Rhizo 3) from the current study.

**Figure 2:**
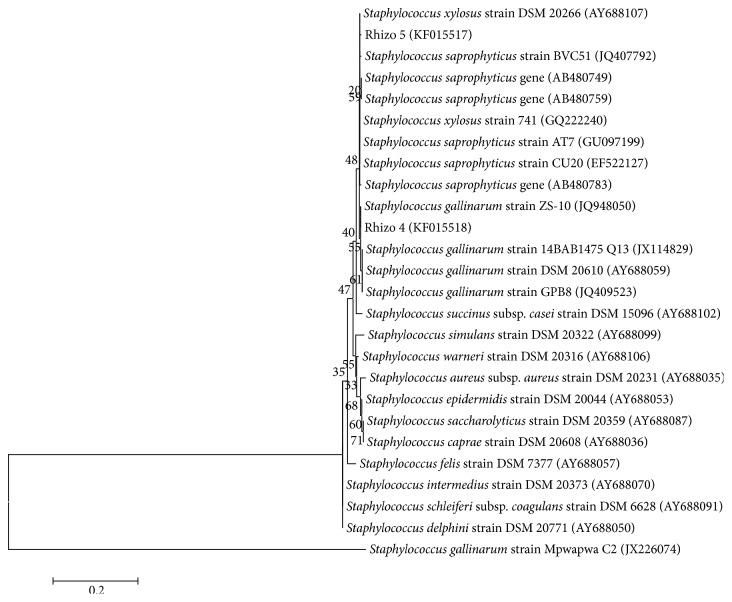
Neighbor-joining (NJ) tree showing the evolutionary relationship of* Staphylococcus gallinarum* (Rhizo 4) and* Staphylococcus saprophyticus* (Rhizo 5) from the current study.

The bacterial isolates were identified based on 16S rDNA sequencing. PCR amplification yielded ~1600 bp amplicon in all the isolates. BLAST analysis of isolates Rhizo 1, Rhizo 2, and Rhizo 3 showed 100% similarity to* Bacillus subtilis *available in the Genbank database and thus the bacterium was identified as* Bacillus subtilis*. Similarly, isolate Rhizo 4 showed 100% similarity to* Staphylococcus gallinarum *sequence and was identified as* Staphylococcus gallinarum. *The isolate Rhizo 5 showed 100% similarity to* Staphylococcus saprophyticus *sequence and was identified as* Staphylococcus saprophyticus.* The sequence data generated from the study was deposited in the Genbank nucleotide database (NCBI) and the accession numbers assigned are as follows: isolate 1: KF015514; isolate 2: KF015515; isolate 3: KF015516; isolate 4: KF015518; isolate 5: KF015517. The bacterial isolates were successfully grouped to their respective reference strains obtained from the Genbank database confirming the authenticity of the isolate (Figures 1 and 2).

### 3.3. Antibiotic Susceptibility

The antibiotic activity against the endosymbiotic bacteria isolated from* R. amorphophalli *is presented in Table 2. All the isolates were resistant to cephalexin and they recorded highest activity against cefixime.

## 4. Discussion

Almost all insects have endosymbionts for their normal growth and development [20]. Loss of these microorganisms often results in abnormal development and reduces survival of the insect host [21]. Previously, many reports described the isolation of bacteria from mealy bugs and other sap sucking insects. However, the present study is the first to identify bacteria associated with mealy bug,* Rhizoecus amorphophalli, *employing phenotypic and 16S rDNA sequence analysis. Three culturable bacteria,* namely, Bacillus subtilis, Staphylococcus gallinarum,* and* Staphylococcus saprophyticus,* were isolated from* R. amorphophalli*. BLAST search for these isolates showed the highest match with the respective species and all the sequences were deposited with NCBI-Genbank.

Isolates 1, 2, and 3 showed similar phenotypic characters. When compared with Bergey's Manual of Systematic Bacteriology (2009) it was seen that the biochemical tests of three isolates showed similarity with* Bacillus *sp. But, there were few exceptions as for* Bacillus *acid will be produced all sugars. However, in our study acid was produced only from dextrose and lactose and all other sugars tested showed negative reaction. Similarly, isolates Rhizo 4 and Rhizo 5 showed biochemical characters of* Staphylococcus *when compared with agreement with Bergey's Manual of Systematic Bacteriology Volume 2 (2005). But present results showed dissimilarity in sugar test and nitrate reduction test when compared with Bergey's Manual.

Classical biochemical identification at the species level was found very difficult and often confusing [22]. A combination of biochemical and molecular methods may prove to be more helpful in obtaining a precise and credible identification for the isolates [23]. Therefore one should associate the results of biochemical test with the molecular methods to confirm the identification of the isolate under study. In the current study, 16S rDNA sequence analysis was performed successfully and bacterial isolates were identified undoubtedly.

Bacteria of genus* Bacillus *and* Staphylococcus *were previously reported from many insect species but, among the three isolates,* S. saprophyticus* was less reported as endosymbiont from insects.* Bacillus subtilis *was previously reported as an endosymbiont of whitefly,* B. tabaci *[24]. From the intestine of silk worm,* Bombyx mori* L., nine bacterial strains belonging to genera* Bacillus, Brevibacterium, Corynebacterium, Staphylococcus, Klebsiella,* and* Stenotrophomonas* were identified [25]. From the gut of* Aedes aegypti *L.,* B. subtilis *and* Serratia* sp. were found [26]. Gram positive bacteria including* B. subtilis* were identified from* Musca domestica* L. [27].

The phenotypic characteristics of 15 probable species of* Bacillus *wereidentified and it was found that they are gram positive rods which were capable of hydrolysing starch and casein [28]. In this study they found that the most dominating species was* B*.* subtilis *when compared with the other species found (*B*.* pumilus *and* B*.* mycoides*).* B. pumilus*, one of the most dominant bacteria of all the populations in insects, is a gram positive, aerobic, rod-shaped, soil-dwelling bacterium. Like other* Bacillus* species, the spores produced by* B. pumilus* are more resistant than vegetative cells to heat, desiccation, UV radiation, *γ*-radiation, H_2_O_2_, and starvation. The presence of this has been previously reported from gut flora of adult* Phlebotomus papatasi*. But* B*.* pumilus *and* B*.* mycoides* are found in mealy bug [29]. Mukhopadhyay et al. [29] reported that* B. cereus* is dominant species in* Phlebotomus papatasi*. This clearly indicated that the dominant bacterial species in insect varies according to species. One important power possessed by* Bacillus *strains isolated is their ability to produce amylase enzyme [30, 31], and these amylases are involved in the initial breakdown of cassava starch into simple sugars. Similarly, the endosymbiont* B*.* subtilis* may be helping the* R. amorphophalli*, by production of amylase enzyme required for conversion of starch to simple sugars. Eleven isolates from the silk worm were identified which included the gram positive* Bacillus circulans *that degrade cellulose, pectin, and starch and have impact on digestion [32].

*Staphylococcus gallinarum* was also reported as endosymbionts from many insect species.* Staphylococcus gallinarum* and* S. saprophyticus *were isolated from B and Q types of* B. tabaci*, respectively [33]. Several gram positive bacteria including* Lactococcus garvieae* and* S. saprophyticus* were isolated from the gut of red imported fire ant,* Solenopsis invicta* Buren, using 16S rDNA sequences [34].* Staphylococcus saprophyticus *was less reported as endosymbionts of insects but was recorded from many living systems [35].* Bacillus* and* Staphylococcus* from the whitefly were reported for their potential to produce medium length sugars from sucrose and contribute to the stickiness of the honeydew secreted by the host insect [33] and these bacteria were recorded from* R. amorphophalli* also.

The bacterial association with arthropods has been used to develop novel control strategies of agricultural, medical, and veterinary important pests [36]. Based on microecology theory, insects lack a complete enzyme system and thus need gut microorganisms to provide different kinds of enzymes for food digestion, nutrient absorption, and biological metabolism [37].

Antibiotic susceptibility tests showed that all the isolates were resistant to the antibiotics except cephalexin. Antibiotic sensitivity pattern of* Bacillus *sp. was studied and results showed that* B. subtilis* was resistant to gentamicin, ampiclox, and zinnacef and susceptible to ciprofloxacin [28].* Staphylococcus saprophyticus* was susceptible to ampicillin, cephalexin, and trimethoprim-sulfamethoxazole and resistant to nalidixic acid and novobiocin [38].

Endosymbionts could be used as effective biocontrol agents (BCAs) and the present study will stand as a connecting link in identification and effective utilization of these endosymbionts as BCAs and also in formulating newer IPM strategy for* R. amorphophalli *by employing these three bacteria.

## Figures and Tables

**(a):** 

Isolate	Colony characters	Gram reaction	Shape	Acid production							ONPG
				Dextrose	Lactose	Sucrose	Mannitol	Maltose	Fructose	Starch	
Rhizo 1	Circular, entirely flat	P	Rod	P	P	N	N	N	N	N	N
Rhizo 2	Circular, entirely flat	P	Rod	P	P	N	N	N	N	N	N
Rhizo 3	Circular, entirely flat	P	Rod	P	P	N	N	N	N	N	N
Rhizo 4	Irregular, creamy yellow, lobate	P	Cocci	P	P	P	P	P	P	P	N
Rhizo 5	Small, irregular, lobate, white	P	Cocci	P	P	P	P	P	P	P	N

**(b):** 

Isolate	Urease	IMViC				TSI	Nitrate production	MIO	Motility	Phenotypic identification	16S rDNA identification
		Indole	MR	VP	Citrate						
Rhizo 1	SP	N	N	N	P	Acidic slant, acidic butt	P	N	Motile	*Bacillus* sp.	*Bacillus subtilis *
Rhizo 2	SP	N	N	N	P	Acidic slant, acidic butt	P	N	Motile	*Bacillus* sp.	*Bacillus subtilis *
Rhizo 3	SP	N	N	N	P	Acidic slant, acidic butt	P	N	Motile	*Bacillus* sp.	*Bacillus subtilis *
Rhizo 4	P	N	P	N	P	Acidic slant, acidic butt	P	N	Motile	*Staphylococcus* sp.	*Staphylococcus gallinarum *
Rhizo 5	P	N	N	N	P	Acidic slant, acidic butt	P	N	Nonmotile	*Staphylococcus* sp.	*Staphylococcus saprophyticus *

P: positive, N: negative, and SP: slightly positive.

**Table 2:** Antibiotic activity against the endosymbiotic bacteria of *Rhizoecus amorphophalli*.

	Antibiotic activity (cm)			
	Cephalexin	Ciprofloxacin	Endrofloxaxin	Cefixime
Rhizo 1	R	3.7	2.2	3.8
Rhizo 2	R	3.4	2.8	4.0
Rhizo 3	R	3.5	2.7	3.8
Rhizo 4	R	2.5	2.3	2.8
Rhizo 5	R	2.8	2.7	3.1

## References

[1] Douglas A. E. (1998). Nutritional interactions in insect-microbial symbioses: aphids and their symbiotic bacteria *Buchnera*. *Annual Review of Entomology*.

[2] Scarborough C. L., Ferrari J., Godfray H. C. (2005). Aphid protected from pathogen by endosymbiont. *Science*.

[3] Douglas A. E. (2007). Symbiotic microorganisms: untapped resources for insect pest control. *Trends in Biotechnology*.

[4] Ben-Dov Y. (1994). *A Systematic Catalogue of the Mealy Bugs of the World*.

[5] Joshi M. D., Butani P. G., Patel V. N., Jeyakumar P. (2010). Cotton mealy bug, *Phenacoccus solenopsis* Tinsley—a review. *Agricultural Review*.

[6] Goldasteh S., Talebi A. A., Fathipour Y., Ostovan H., Zamani A., Shoushtari R. V. (2009). Effect of temperature on life history and population growth parameters of Planococcus citri (Homoptera, Pseudococcidae) on coleus [Solenostemon scutellarioides (L.) Codd.]. *Archives of Biological Sciences*.

[7] Galanihe L. D., Jayasundera M. U. P., Vithana A., Asselaarachchi N., Watson G. W. (2010). Occurrence, distribution and control of papaya mealy bug, *Paracoccus marginatus* (Hemiptera: Pseudococcidae), an invasive alien pest in Sri Lanka. *Tropical Agricultural Research and Extension*.

[8] Ramanandam G., Ravisankar C., Srihari D. (2008). Integrated nutrient management of cassava under rain fed conditions of Andhra Pradesh. *Journal of Root Crops*.

[9] Quaye W., Gayin J., Yawson I., Plahar W. A. (2009). Characteristics of various cassava processing methods and the adoption requirements in Ghana. *Journal of Root Crops*.

[10] Palaniswami M. S., Chadha K. L., Nayar G. G. (1994). Pests of edible aroids, yams and Chinese potato. *Advances in Horticulture, Vol. 8, Tuber crops*.

[11] Palaniswami M. S., Tarafdar J., Palaniswami M. S., Peter K. V. (2008). Pests and diseases: their management and plant quarantine. *Tuber and Root Crops*.

[12] Sanchez-Contreras M., Vlisidou I. (2008). The diversity of insect-bacteria interactions and its applications for disease control. *Biotechnology and Genetic Engineering Reviews*.

[13] Hirose E., Panizzi A. R., de Souza J. T., Cattelan A. J., Aldrich J. R. (2006). Bacteria in the gut of southern green stink bug (Heteroptera: Pentatomidae). *Annals of the Entomological Society of America*.

[14] Mackie T. J., Collee J. G., McCartney J. E. (1989). *Mackie & McCartney Practical Medical Microbiology*.

[15] Leroy P. D., Sabri A., Heuskin S. (2011). Microorganisms from aphid honeydew attract and enhance the efficacy of natural enemies. *Nature Communications*.

[16] Tamura K., Peterson D., Peterson N., Stecher G., Nei M., Kumar S. (2011). MEGA5: molecular evolutionary genetics analysis using maximum likelihood, evolutionary distance, and maximum parsimony methods. *Molecular Biology and Evolution*.

[17] Thompson J. D., Higgins D. G., Gibson T. J. (1994). CLUSTAL W: improving the sensitivity of progressive multiple sequence alignment through sequence weighting, position-specific gap penalties and weight matrix choice. *Nucleic Acids Research*.

[18] Saitou N., Nei M. (1987). The neighbor-joining method: a new method for reconstructing phylogenetic trees. *Molecular Biology and Evolution*.

[19] Bauer A. W., Kirby W. M., Sherris J. C., Turck M. (1966). Antibiotic susceptibility testing by a standardized single disk method. *The American Journal of Clinical Pathology*.

[20] Munson M. A., Baumann P., Morant N. A. (1992). Phylogenetic relationships of the endosymbionts of mealybugs (Homoptera: Pseudococcidae) based on 165 rDNA sequences. *Molecular Phylogenetics and Evolution*.

[21] Fukatsu T., Hosokawa T. (2002). Capsule-transmitted gut symbiotic bacterium of the Japanese common plataspid stinkbug, megacopta punctatissima. *Applied and Environmental Microbiology*.

[22] Thompson J. M., Dodd C. E. R., Waites W. M. (1993). Spoilage of bread by *Bacillus*. *International Biodeterioration & Biodegradation*.

[23] Erem R., Certel M., Karakas B. (2009). Identification of *Bacillus* species isolated from ropey breads both with classical methods and Api identification kits. *Akdeniz Universitesi Ziraat Fakultesi Dergisi*.

[24] Ateyyat M. A., Shatnawi M., Al-Mazra'awi M. (2010). Isolation and identification of culturable forms of bacteria from the sweet potato whitefly *Bemesia tabaci* genn. (Homoptera: Aleyrodidae) in Jordan. *Turkish Journal of Agriculture and Forestry*.

[25] Feng W., Wang X. Q., Zhou W., Liu G. Y., Wan Y. J. (2011). Isolation and characterization of lipase-producing bacteria in the intestine of the silkworm, *Bombyx mori* reared on different forage. *Journal of Insect Science*.

[26] Gusmão D. S., Santos A. V., Marini D. C., Bacci M., Berbert-Molina M. A., Lemos F. J. A. (2010). Culture-dependent and culture-independent characterization of microorganisms associated with Aedes aegypti (Diptera: Culicidae) (L.) and dynamics of bacterial colonization in the midgut. *Acta Tropica*.

[27] Banjo A. D., Lawal O. A., Adeduji O. O. (2005). Bacteria and fungi isolated from housefly (*Musca domestica* L.) larvae. *African Journal of Biotechnology*.

[28] Adewumi G. A., Quadri R. A., Oguntoyinbo F. A. (2009). Antibiotic sensitivity pattern of *Bacillus* species isolated from solid substrate fermentation of cassava for gari production. *African Journal of Microbiology Research*.

[29] Mukhopadhyay J., Braig H. R., Rowton E. D., Ghosh K. (2012). Naturally occurring culturable aerobic gut flora of adult *Phlebotomus papatasi*, vector of *Leishmania major* in the old world. *PLoS ONE*.

[30] Amund O. O., Ogunsina O. A. (1987). Extracellular amylase production by cassava-fermenting bacteria. *Journal of Industrial Microbiology*.

[31] Oyewole O. B., Odunfa S. A. (1992). Extracellular enzyme activities during cassava fermentation for ‘fufu’ production. *World Journal of Microbiology & Biotechnology*.

[32] Anand A. A. P., Vennison S. J., Sankar S. G. (2010). Isolation and characterization of bacteria from the gut of *Bombyx mori* that degrade cellulose, xylan, pectin and starch and their impact on digestion. *Journal of Insect Science*.

[33] Indiragandhi P., Yoon C., Yang J. O., Cho S., Sa T. M., Kim G. H. (2010). Microbial communities in the developmental stages of B and Q biotypes of sweetpotato whitefly, *Bemisia tabaci* (hemiptera: Aleyrodidae). *Journal of Applied Biological Chemistry*.

[34] Peloquin J. J., Greenberg L. (2003). Identification of midgut bacteria from fourth instar red imported fire ant larvae, *Solenopsis invicta* buren (Hymenoptera: Formicidae). *Journal of Agricultural and Urban Entomology*.

[35] Goja A. M., Ahmed T. A. A., Saeed S. A. M., Dirar H. A. (2013). Isolation and identification of *Staphylococcus* spp. in fresh beef. *Pakistan Journal of Nutrition*.

[36] Broderick N. A., Raffa K. F., Goodman R. M., Handelsman J. (2004). Census of the bacterial community of the gypsy moth larval midgut by using culturing and culture-independent methods. *Applied and Environmental Microbiology*.

[37] Wei X. (1985). *Normal Flora and Health*.

[38] Marrie T. J., Kwan C. (1982). Antimicrobial susceptibility of *Staphylococcus saprophyticus* and urethral staphylococci. *Antimicrobial Agents and Chemotherapy*.

